# Adults show selective responses to unreliability based on the strength of counterevidence

**DOI:** 10.1371/journal.pone.0331480

**Published:** 2025-11-13

**Authors:** Kirsten H. Blakey, Giacomo Melis, Zsófia Virányi, Eva Rafetseder

**Affiliations:** 1 Philosophy, Faculty of Arts and Humanities, University of Stirling, United Kingdom; 2 Psychology, Faculty of Natural Sciences, University of Stirling, United Kingdom; 3 Psychological & Brain Sciences, University of Toronto Mississauga, Canada; 4 Comparative Cognition, Messerli Research Institute, University of Veterinary Medicine, Vienna, Medical University of Vienna, University of Vienna, Austria; RIKEN CBS: RIKEN Noshinkei Kagaku Kenkyu Center, JAPAN

## Abstract

Adults can reflectively revise their beliefs and selectively respond to unreliable informants, despite often forming and revising beliefs unreflectively without assessing their reasons. This study investigates how the strength of counterevidence coming from an informant affects adults’ ability to infer that the informant is unreliable through acquiring and responding to undermining defeaters (i.e., evidence suggesting that something was wrong with how the belief was formed). Participants (*N *= 120) watched videos of two informants acting on two locations: one whose actions reliably indicated the reward location, and one whose actions did not. The strength of feedback participants received after making a choice was manipulated across two conditions. In the Strong feedback condition, participants received positive feedback when they found the reward and explicit negative feedback when they did not, along with information about the reward’s true location. In the Weak feedback condition, they received positive feedback, but incorrect choices simply resulted in no reward. Participants responded selectively to unreliability, following the Unreliable informant’s evidence less often than that of the Reliable informant. This effect was stronger in the Strong feedback condition and was observed after only two to three misleading trials. In subsequent trials where informants were pitted against each other, participants in the Strong feedback condition, but not in the Weak feedback condition, consistently preferred the Reliable informant. These findings suggest that adults’ ability to infer informants’ reliability depends on the strength of counterevidence. Additionally, exploratory analyses reveal a key distinction between acquiring and responding to undermining defeaters.

## Introduction

In daily life, we constantly encounter information that shapes our beliefs—whether from news, social media, or personal experiences. Sometimes the information we gather from such sources is false or misleading. But how often do we pause to question the reliability of these sources? And how do we come to trust a specific source of information and not another? Young children [1–3] and animals [4,5] prefer reliable or accurate informants over unreliable ones. However, it is often unclear whether they are identifying and assessing their reasons for belief or simply relying on associations or heuristic biases [6–8]. In contrast, adults are capable of reflectively assessing the information they gather, although they can still be misled by unreliable sources, as seen in the widespread presence of conspiracy theories. Still, in principle adults can identify and assess the accuracy of the information received, and the reliability of the source that delivered it, thereby recognising when evidence coming from an unreliable source may be misleading. For example, a person may initially trust a news story because it comes from a reputable outlet. However, upon discovering that the journalist has a history of fabricating data, they could reassess the credibility of the source and adjust their belief about the story.

Despite their capacity for assessing the reliability of a source, adults often form and revise beliefs in their daily lives without consciously identifying and assessing the evidence for doing so [9–11]. For instance, one might believe that their keys are on the table based on their memory of placing them there. However, upon checking the table and discovering that the keys are not there, one is likely to revise their belief based on this new perceptual evidence, a process that typically occurs automatically and unreflectively in response to the *overriding defeater*. Overriding defeaters suggest that one should replace their belief in a proposition P <the keys are on the table> with its negation, not-P <the keys are not on the table>, which can be done unreflectively because it does not require assessment of evidence. However, even in such cases, adults can generally articulate their reasons for or the evidence behind both their current and past beliefs if asked to do so.

Some philosophers contend that the ability to articulate reasons for a belief is the mark of rationality [12–14] and that unreflective responsiveness to evidence alone is insufficient for rational belief formation or revision [15,16]. In contrast, others allow that unreflective responsiveness to evidence is sufficient for rationality, and can even be found in non-linguistic populations such as non-human animals and pre-verbal children [17–21]. To bridge these two positions, it is important to understand the relationship between the reflective belief revision we observe in adults and the unreflective belief revision that appears to be present both in adults and non-linguistic populations. Key to this is understanding how overriding counterevidence, processed automatically, comes to make a subject identify and assess a source of evidence as being unreliable.

To assess the transition from unreflective to reflective belief revision we focus on a particular type of counterevidence known as undermining defeaters. Undermining defeaters are reasons to reduce or give up confidence in one’s belief, typically suggesting that something is wrong with how the belief was formed, for example <the source of the evidence is unreliable> [22,23]. Responding to an undermining defeater is considered reflective as it typically involves: 1) identifying a piece of information as evidence, and 2) assessing that evidence as being potentially misleading [24], both hallmarks of reflective belief revision [14]. While undermining defeaters are often acquired through testimony, they can also be acquired in other ways. For instance, undermining defeaters can be acquired by making an inference over repeated responses to overriding defeaters regarding beliefs formed following the evidence coming from the same source (e.g., an unreliable person). Experiencing several overriding defeaters coming from the same source supports the inference that <the source is unreliable>. Making such an inference indicates that an undermining defeater has been acquired. Once acquired, responding to the undermining defeater should lead to refusal—or reduced willingness—to follow new evidence provided by the same unreliable source, even in different contexts.

Recent empirical research demonstrates that it is possible to empirically investigate responses to non-verbal overriding defeaters in young children and great apes [25] and responses to undermining defeaters in 4- to 5-year-old children using testimonial evidence [26]. However, only one study has investigated whether undermining defeaters can be acquired via inference over repeated responses to overriding defeaters. Blakey et al. [27] put 2-year-old children, dogs, and pigs in the position to acquire and respond to an undermining defeater regarding informants’ reliability. In this task, subjects observed informants act on two screens. One informant reliably indicated the reward location, while the other only indicated the reward location 50% of the time. Each time the Unreliable informant misled subjects, they were expected to respond to an overriding defeater, replacing their belief <the reward is in the location where the person acted> with the belief <the reward is *not* in the location where the person acted>. Repeated exposure to these overriding defeaters was intended to lead subjects to infer an undermining defeater related to the informant, such as <this informant is unreliable>. Consequently, in a new context where informants used a new action to indicate a screen, subjects should have been less willing to follow the Unreliable informant’s evidence relative to the Reliable informant’s evidence. However, results revealed that neither children nor animals differentiated between the Reliable and the Unreliable informants, as they followed the evidence from both informants less frequently in new contexts. One interpretation is that they may have acquired and responded to an undermining defeater, such as <both informants are unreliable>. Alternatively, they may have been responding to their growing uncertainty about what to do.

One possible explanation for participants’ failure to identify the Unreliable informant in Blakey et al. [27] is that the overriding evidence (the reward not being in the indicated location) might not have been strong enough to warrant the generalisation to <this informant is unreliable> and thereby acquisition of an undermining defeater. In general, children are sensitive to the strength of evidence and counterevidence. For example, 4- and 5-year-olds are more likely to revise their beliefs when their initial belief is based on weak evidence and they encounter stronger counterevidence [26,28]. Even children as young as 3 years old have demonstrated belief revision influenced by the evidential strength of informants’ claims, with revisions happening more often when claims are based on direct (first-person) observation and inference (evidence-based) rather than on indirect (third-person) observation [29]. Although this shows that children do use overriding evidence to revise their beliefs, they may struggle to leverage the cumulative strength of overriding defeaters as a foundation for acquiring an undermining defeater. Therefore, to justify the generalisation that an informant, who initially supported the belief, is actually unreliable, a sufficient level of strength in the cumulative overriding evidence seems essential. Understanding how the accumulation of such strength occurs is also an important element in accounting for how counterevidence initially processed automatically eventually makes a subject aware of the unreliability of a source of information. As adults can assess informant reliability, exploring how they acquire and respond to undermining defeaters in non-verbal contexts promises to enhance the development of paradigms for evaluating this ability in young children and animals.

The current study investigates how varying the strength of overriding evidence affects adults’ ability to acquire and respond to an undermining defeater. To achieve this, we adapted the task developed by Blakey et al. [27] for use on a tablet, incorporating videos of informants while preserving the task’s original structure. The strength of overriding evidence was manipulated across two conditions by varying the quantity and quality of feedback received after making a choice (Fig 2). In the Weak feedback condition, choosing the correct location revealed the reward, while choosing the incorrect location resulted in no reward. In the Strong feedback condition, additional cues were provided: a green check mark indicated a correct choice, and a black X marked an incorrect choice, with the reward appearing in the alternate location after a brief delay. In terms of *quantity*, the Strong feedback condition provided two pieces of evidence per choice: for correct choices, participants saw both the reward and a green check mark (both positive evidence); for incorrect choices, they saw both an X and the reward appearing above the unselected location (both overriding evidence). In contrast, the Weak feedback condition offered only one piece of evidence—either the presence or absence of the reward (i.e., positive or overriding evidence, respectively). For *quality*, in the Strong feedback condition, the two pieces of positive evidence had two distinct contents for correct choices: the presence of the reward and a green check mark. For incorrect choices, the two pieces of overriding evidence combined the reward’s absence with a black X. In the Weak feedback condition, appearance of the reward in the selected location represented positive evidence, while absence of the reward provided overriding evidence for incorrect choices. The combined variation of the quantity and quality of evidence highlights a difference in the strength of both positive and overriding evidence participants received in the two conditions.

**Fig 1:**
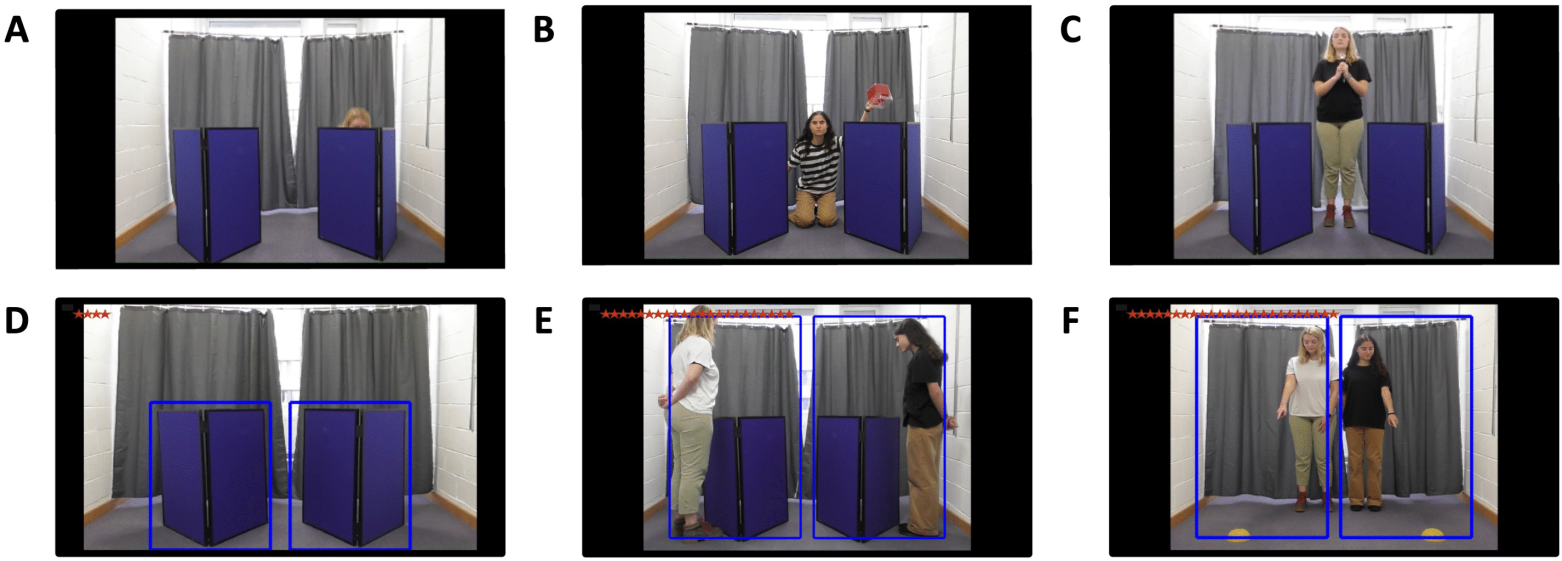
Demonstration actions and choice screens for each trial type. Panels **A–C** depict the different action types presented during the demonstration trials: **A.** Crouching, **B.** Lifting, and **C.** Sound. Panels **D–F** show the different choice screens used in: **D.** Demonstration trials. **E.** Screen Choice Transfer trials, and **F.** Pointing Transfer trials.

We expected that participants would respond selectively to the reliability of the informants, with evidence from the Unreliable informant being followed less often than that from the Reliable informant, after being misled several times by the Unreliable informant. Additionally, we predicted that, if the strength of the evidence is crucial for acquiring an undermining defeater, stronger feedback would enhance the justification for overriding initial beliefs, making participants more likely to acquire and respond to an undermining defeater in the Strong feedback condition. The more overriding evidence participants receive, particularly when that overriding evidence varies in content, the stronger the basis may be for generalising the informant’s unreliability.

## Method

### Participants

The final sample included 120 participants (84 females, 35 males, 1 AFAB; *M*_age_ = 21.2 years, SD = 7 years, range = 17–58 years). Participants predominantly identified themselves as British. Participants were randomly allocated to the Strong feedback condition (43 females, 16 males, 1 AFAB; *M*_age_ = 20.3 years, SD = 5.3 years) or the Weak feedback condition (41 females, 19 males; *M*_age_ = 21.9 years, SD = 8.3 years). Participants were university students recruited at the University of Stirling between 17 October 2023 and 16 November 2023. Participation was voluntary and students received research participation tokens towards course completion. Three additional participants were excluded due to technical problems. The sample size of 60 participants per condition was preregistered based on conventions in the literature, aiming to achieve approximately 80% power to detect a medium effect size.

This study was approved by the General University Ethics Panel at the University of Stirling (2023 3029 10554). Research was conducted in accordance with relevant guidelines and regulations. Informed consent was obtained via an online survey, which included the participant information sheet. Participants provided written electronic consent prior to participation by selecting an option to confirm agreement.

### Equipment

Participants completed the task on a touchscreen Microsoft Surface tablet wearing Klugmia over-ear headphones. The experiment was written and run using PsychoPy v2023.2.2 [30]; the task code and stimuli are available on the OSF: https://osf.io/xhe8t/.

### Design and procedure

Participants were presented with on-screen instructions explaining that they were searching for rewards and had 5 seconds to make each choice. They then completed a practice trial in which a blue V-shaped screen was surrounded by a blue rectangular box, along with the instruction *“Let’s practice how to look for a reward. Swipe inside the blue box to look for a reward.”*

The task included 44 Demonstration trials and six Transfer trials (for detailed order see S1 Table). The demonstration trials allowed participants to infer informant reliability through repeated experiences and to recognise that different forms of evidence from the same source can be misleading, thereby acquiring an undermining defeater. The transfer trials tested whether this undermining defeater could be applied in increasingly different contexts, by examining preferences when informants were pitted against one another.

In demonstration trials, participants watched a video of an informant holding a reward (red star) and using an action (crouching, lifting, or sound) to indicate one of two possible locations (blue V-shaped screens). The actions involved crouching behind one of the screens (Fig 1A), lifting a red coloured box above one of the screens (Fig 1B), or standing between the screens ringing a bell that could be heard through one side of the headphones (Fig 1C). After watching the video, an image of the two screens each within a blue rectangular box appeared, and participants could choose one location to search for the reward (Fig 1D).

In transfer trials, participants chose between the Reliable and Unreliable informants who were presented together. In four screen choice trials each informant was positioned at the outer side of a screen (Fig 1E), while in two pointing trials the informants stood next to each other and pointed and gazed at one of two upturned bowls (Fig 1F). In both cases the informant and the corresponding screen or bowl were surrounded by a blue box.

Three female actors played the roles of Demonstrator, Reliable informant, and Unreliable informant (wearing different coloured shirts: black, white, and striped). The Demonstrator and the Reliable informant always indicated the reward location, while the Unreliable informant did so in only 50% of trials, and never on the first trial of each action. Analyses focused on responses to the Reliable and Unreliable informants and accounted for variations in actor pairings.

Before the first trial, participants saw all three actors (Demonstrator in the centre) for 5 seconds. The screen that was acted on and whether the Unreliable informant indicated the rewarded location were pseudorandomised, with no more than two consecutive trials with the same action, reward side, or misleading cue. The role played by each actor was randomised, with two sets of videos providing two possible shirt colours per actor. Within subjects, informant role and shirt colour were fixed, but this varied between subjects.

**Fig 2:**
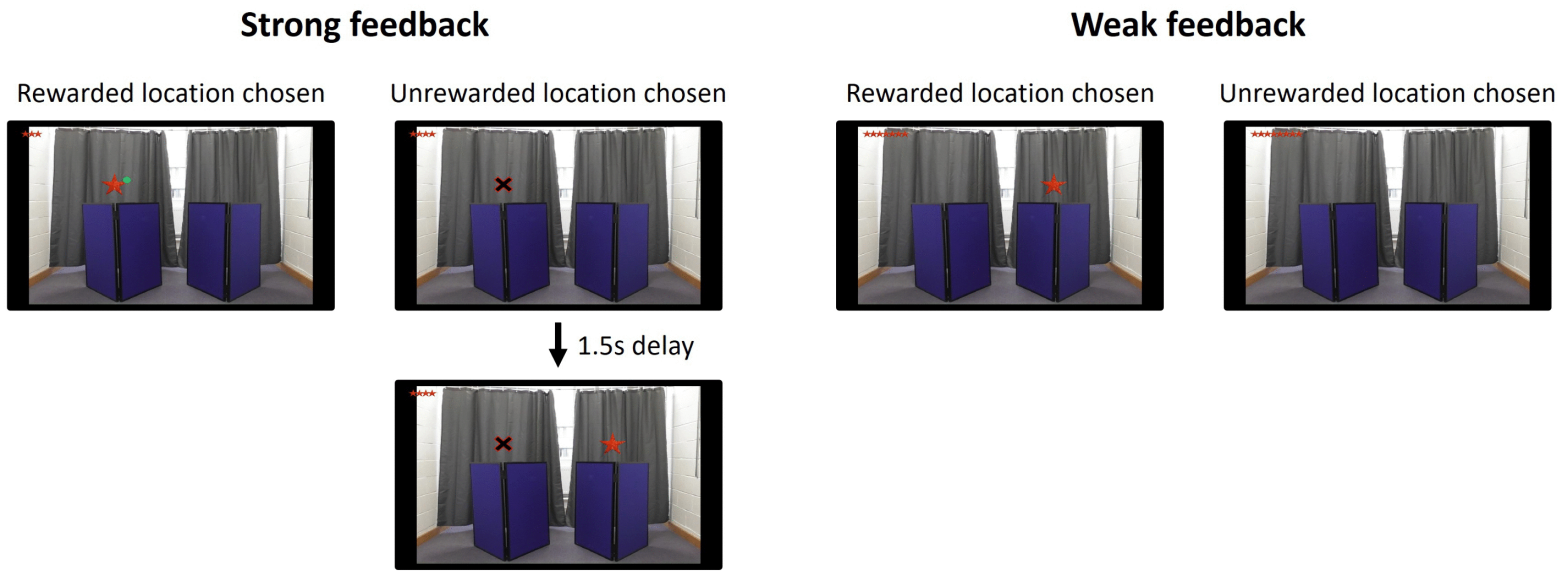
Feedback in Strong and Weak feedback conditions. Illustration of the feedback received in each condition when rewarded and unrewarded locations were chosen.

There were two conditions: the Strong feedback condition and the Weak feedback condition. These differed only in the quantity (pieces of positive and overriding evidence) and quality (content of positive and overriding evidence) of evidence participants received after making a choice. In the Strong feedback condition, choosing the rewarded location caused the reward and a small green check mark to appear above the corresponding screen (Fig 2). If the unrewarded location was chosen, a black X with a red outline appeared above the selected screen and after 1.5 seconds the reward (a red star) appeared above the other screen (without a green check mark). In the Weak feedback condition, choosing the rewarded location caused the reward (without a green check mark) to appear above the corresponding screen. If the unrewarded location was chosen, there was no explicit sign that the reward had not been found and no indication that the alternative location was rewarded. In both conditions trials timed out after 5 seconds. A reward bar at the top of the screen was updated on every trial to show how many rewards had been found. The task lasted approximately 12 minutes.

### Statistical analysis

All data have been made publicly available on the OSF and can be accessed at https://osf.io/xhe8t/. This study’s design and analyses were preregistered prospectively, before data were collected; see https://doi.org/10.17605/OSF.IO/B9P6Q. Deviations from the preregistration are indicated as such or described as exploratory.

All analyses were performed using R [31]. For analysing the demonstration trials, we fit generalised linear mixed effects models (GLMMs) using *lme4* [32] with logit regression. The dependent variable was participants’ probability to follow the evidence, and included fixed effects of informant (Reliable, Unreliable), action number (Crouching –1, Lifting 0, Sound 1), condition (Strong feedback, Weak feedback), the interactions between these variables, and a control fixed effect of action side (left, right). For Transfer trial analysis, we fit a GLMM with preferred informant as the dependent variable (Unreliable 0, Reliable 1), including fixed effects of condition (Strong feedback, Weak feedback), transfer task (Screen choice, Pointing), trial number within task, the interaction between these variables, and a control fixed effect of Reliable informant side (left, right). To account for repeated observations, we included participant ID as a random intercept with random slopes for all fixed effects, keeping random effects structures ‘maximal’ [33]. We also included colour-dyad (pairing of informants’ reliability and colour assignment) as a random intercept with random slopes for all fixed effects. Where specified as fixed effects, the following variables were dummy-coded and centred: informant, condition, action side, and Reliable informant side.

Collinearity among predictor variables was assessed by calculating Variance Inflation Factors (VIFs) for standard linear models, excluding random effects and interactions, using the *car* package [34]. Collinearity was not an issue in the Demonstration trial model (VIFs = 1) or the Transfer trial model (VIFs ≤ 1.5). To avoid issues of multiple testing, we assessed the overall effect of the predictors of interest by comparing the full model with a null model lacking these predictors [35] using a likelihood ratio test [36]. Fixed effects and interactions were then individually tested by dropping them one at a time from the model and comparing the models using likelihood ratio tests [33]. Post hoc analyses were carried out using estimated marginal means using the *emmeans* package [37]. P values < .05 were accepted as statistically significant.

## Results

The data and code necessary to reproduce the analyses presented are available on OSF: https://osf.io/xhe8t/.

### Demonstration trials

**Fig 3:**
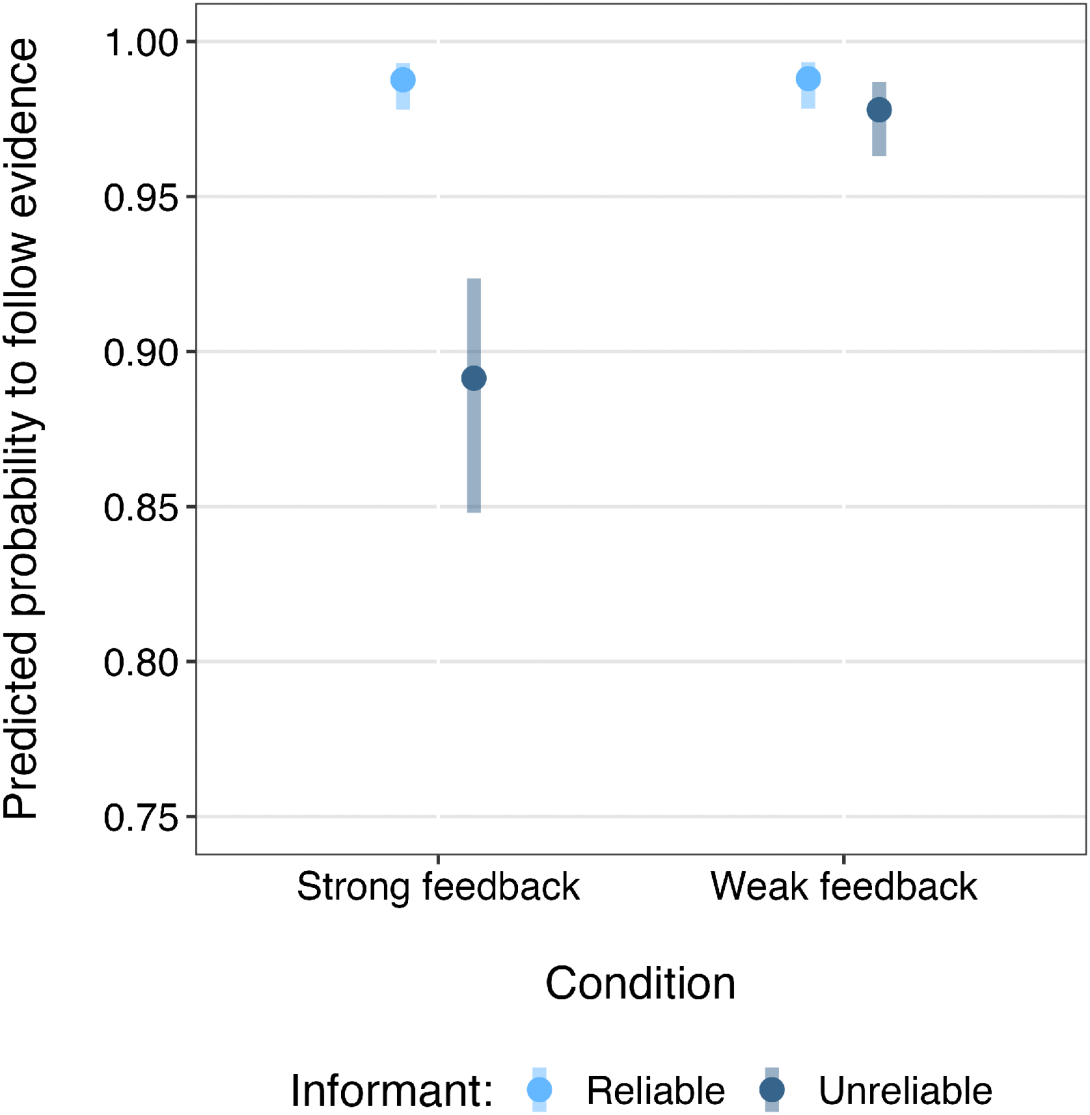
Predicted probability of following each informant. Predicted probability of participants in each condition following the evidence provided by each informant. Results are averaged over the effects of action side. Confidence intervals (95%) are shown for each informant-condition combination. To allow the confidence intervals to be more visible, the y-axis is restricted to the range 0.75–1 (rather than the full 0–1). The corresponding plot with the full scale is shown in Fig S1 in S1 File.

The binomial GLMM for evidence following in the full data set was significantly better than the null equivalent (*χ*^2^(7) = 46.61, *p* < .001). There was no evidence of a three-way interaction between informant, action number, and condition, and comparison with a reduced model without the interaction showed no significant difference (*χ*^2^(1) = 1.74, *p* = .187). Therefore, we examined all two-way interactions, which revealed a significant two-way interaction between informant and condition (*χ*^2^(1) = 10.663, *p* = .001), but no significant interactions involving action number (*p* ≥ .477), therefore they were removed. The final model showed a significant main effect of action number (*b *= –0.42, *SE *= 0.09, *z *= –4.51, *p *< .001) indicating lower evidence following in later actions. There was also a significant interaction between informant and condition (*b *= 1.67, *SE *= 0.39, *z *= 4.31, *p *< .001; Fig 3). Post hoc comparisons revealed that participants were significantly more likely to follow the evidence of the Reliable informant compared to the Unreliable informant in both the Strong feedback (*b *= 2.28, *SE *= 0.26, *z *= 8.81, *p *< .001) and Weak feedback conditions (*b *= 1.28, *SE *= 0.26, *z *= 2.19, *p *= .029). However, they were significantly less likely to follow the evidence of the Unreliable informant in the Strong feedback condition than in the Weak feedback condition (*b *= –1.69, *SE *= 0.31, *z *= –5.48, *p *< .001). In contrast, no significant difference was found between the conditions for the Reliable informant (*b *= –0.03, *SE *= 0.39, *z *= –0.08, *p *= .937).

We intended to run a second GLMM for evidence following in the first trials with each informant in each action, however there were very few trials in which participants did not follow the evidence which made this analysis inappropriate. In both conditions all participants followed the evidence in the first Crouching trials with both the Reliable and Unreliable informants. In the first Lifting and Sound trials there were only 16 (6.7%) and 14 (5.8%) trials across both informants, respectively, in which participants did not follow the evidence.

### Transfer trials

**Fig 4:**
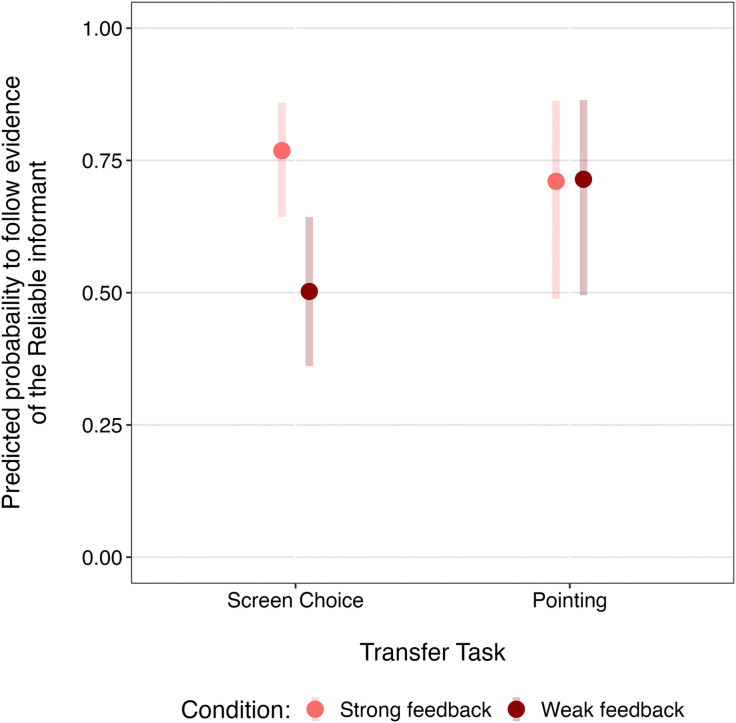
Predicted probability of following the reliable informant. Predicted probability of following the evidence of the Reliable informant in each transfer task and condition. Confidence intervals (95%) are shown for each task-condition combination. Results are averaged over the effects of Reliable informant side.

The GLMM constructed for participants probability to follow the evidence of the Reliable over the Unreliable informant in the transfer trials was significantly better than the equivalent null model (*χ*^2^(7) = 15.35, *p* = .032). There was no evidence of a three-way interaction between condition, task, and task trial number (*χ*^2^(1) = 0.81, *p* = .370), and comparison with a reduced model without the interaction showed no significant difference. Therefore, we examined all two-way interactions, which revealed a significant two-way interaction between condition and task (*b *= 0.59, *SE *= 0.25, *z *= 2.33, *p *= .020; Fig 4). There were no significant interactions or main effects related to task trial number (*p* ≥ .072). Post hoc comparisons of the interaction between condition and task showed that in the screen choice trials, participants in the Strong feedback condition were significantly more likely to follow the evidence provided by the Reliable informant than those in the Weak feedback condition (*b *= 1.19, *SE *= 0.36, *z *= 3.32, *p *< .001), while there was no difference between conditions in the pointing trials (*b *= –0.02, *SE *= 0.44, *z *= –0.04, *p *= .966).

The total number of trials in which participants followed the evidence of the Reliable informant over the Unreliable informant in each transfer task was compared to chance (50%) using one-sample t-tests. Participants in the Strong feedback condition selected the Reliable informant significantly above chance in both the screen choice *t*(198) = 5.78, *p* < .001 and pointing trials *t*(117) = 4.23, *p* < .001. By contrast, participants in the Weak feedback condition only showed a significant preference for the Reliable informant in the pointing trials *t*(117) = 2.34, *p *= .011, not the screen choice trials *t*(193) = –1.65, *p *= .950.

### Response patterns

**Fig 5:**
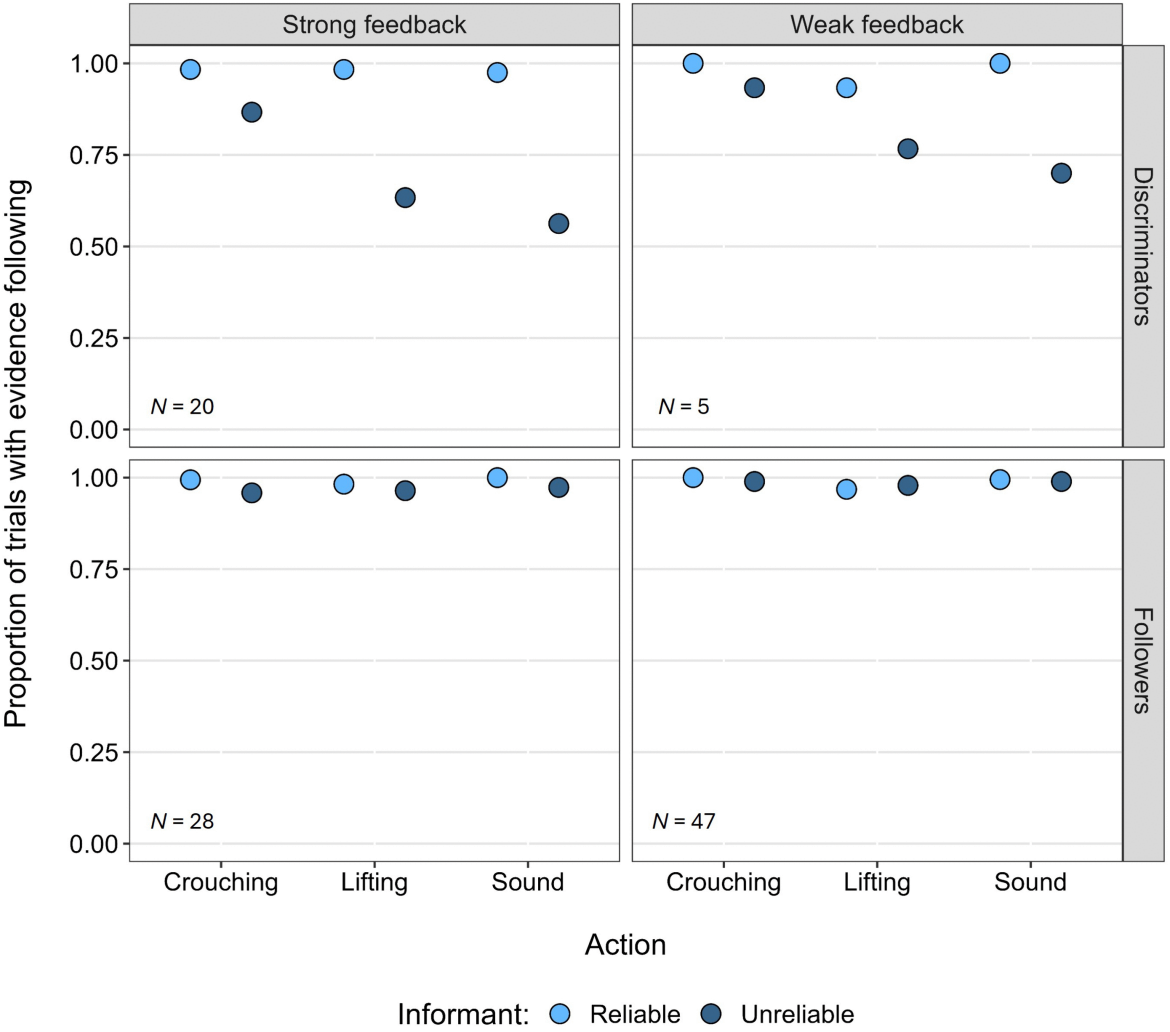
Mean proportion of evidence-following in demonstration trials by response category. Mean proportion of demonstration trials in which participants followed the evidence from each informant, split by condition and response category.

In an exploratory analysis of the *demonstration trials*, we categorised participants according to the pattern of their evidence following with each informant across actions. First, we set out criteria for participants who discriminated between the informants across the actions. To be categorised as “Discriminators”, participants had to have followed the evidence of the Reliable informant ≥75% of trials in each action *and* have followed the evidence of the Unreliable informant ≥66% of the crouching trials, ≤ 84% of the Lifting trials, and ≤75% of the Sound trials (Fig 5 top panels). These criteria capture an initial tendency to follow the informants in crouching trials (with occasional misleading trials) and a growing tendency to choose locations that were not acted on in the lifting and sound trials for the Unreliable informant (these criteria are explained further in S1 File). Participants who followed the evidence of both informants in ≥75% of trials in each action were categorised as “Followers” (Fig 5 bottom panels). The remaining participants – “Others” – did not fit into either of these categories (see S4 Fig in S1 File for individual response patterns). There were 20 Discriminators and 28 Followers in the Strong feedback condition, while in the Weak feedback condition there were 5 Discriminators and 47 Followers. A chi-square test of independence showed a significant association between condition (Strong feedback and Weak feedback) and categories (Discriminators and Followers), *χ*^2^(1) = 22.06, *p* < .001.

For each response pattern within each condition, we conducted one-sample t-tests to assess whether the number of transfer trials in which participants followed the Reliable informant’s evidence (as opposed to the Unreliable informant’s) differed from chance level (50%). In the Strong feedback condition, Discriminators followed the Reliable informant’s evidence in an average of 3.85 transfer trials, significantly above chance *t*(19) = 2.20, *p* = .020. Followers in this condition also selected the Reliable informant significantly above chance in an average of 3.79 transfer trials *t*(27) = 2.82, *p* = .004. In the Weak feedback condition, only Discriminators chose the Reliable informant significantly above chance *t*(4) = 2.45, *p* = .030, averaging 3.60 transfer trials. Followers in the Weak feedback condition, as well as participants in the “Others” group in both conditions, did not differ significantly from chance (*p* ≥ .380).

## Discussion

The current study investigated how the strength of overriding evidence affected adults’ ability to acquire and respond to undermining defeaters in a non-verbal task. Specifically, we manipulated the quantity and quality (content) of evidence that participants received in a Strong feedback condition and a Weak feedback condition. We aimed to determine whether participants could identify and assess evidence from an unreliable informant as being misleading, thereby acquiring an undermining defeater like <this informant is unreliable>. Participants’ responses to the undermining defeater were expected to manifest as a decreased willingness to follow the Unreliable informant’s evidence, while maintaining trust in the Reliable informant’s evidence in subsequent contexts.

The strength of the overriding evidence significantly influenced participants’ willingness to follow the informants’ evidence. Participants in the Strong feedback condition were less likely to follow the Unreliable informant’s evidence than those in the Weak feedback condition, while there was no such difference in following the Reliable informant’s evidence. Contrary to our predictions—and unlike the decrease in following the evidence coming from both informants observed in children and animals [27]—participants reduction in evidence following across the demonstration trials was minimal (albeit significant). It appears that even within the first context (crouching action), participants in the Strong feedback condition were already differentiating between the informants. We found that in the Strong feedback condition it only took two to three trials of being misled by the Unreliable informant for participants to acquire an undermining defeater related to that informant, such as <that source is unreliable>. Consequently, they were already responding to this undermining defeater and continued to do so in subsequent contexts. In contrast, participants showed no indication of acquiring or responding to an undermining defeater in the Weak feedback condition until the end of all three actions.

Given that adults are capable of reflectively revising their beliefs in response to unreliable informants, it is intriguing that the strength of overriding evidence seems to have had such an influence on their responses. Participants in the Strong feedback condition showed clear selectivity in response to unreliability, following the Unreliable informant’s evidence less than that of the Reliable informant, as one would predict for adults. By contrast, in the Weak feedback condition, participants showed far less differentiation between the informants and were more likely to follow the Unreliable informant’s evidence compared to those in the Strong feedback condition. The disparity between the conditions suggests that—consistent with [38]—acquiring an undermining defeater based on previously acquired overriding defeaters requires the overriding evidence supporting the revised belief <the reward is *not* in the location where the person acted> to be markedly stronger than the evidence supporting the initial belief <the reward is in the location where the person acted>. Although the overriding evidence in the Weak feedback condition also speaks in favour of revising the initial belief, it did so more subtly, with only a minority of participants demonstrating recognition of the informants’ reliability. In other words, the additional evidence participants received in the Strong feedback condition appears to have played a crucial role in overriding the initial belief. This brings us to consider what aspects of the evidence in the Strong feedback condition contribute to its greater strength.

Participants in the Strong feedback condition received both a greater quantity of feedback—through additional cues such as a green check mark for finding the reward and an X indicating they missed it, with the reward then appearing in the other location—and a qualitative difference in the nature of this feedback. Following incorrect choices, in the Weak feedback condition, the reward was simply absent, while in the Strong feedback condition the black X explicitly marked the absence of the reward, making the failure more explicit. Participants may have interpreted the X as higher-order evidence indicative of having made a mistake. If so, the X would not necessarily have been stronger than the overriding evidence in the Weak feedback condition (absence of the reward); rather, it would have reinforced the overriding evidence with additional higher-order evidence (i.e., reinforcing that the choice was incorrect). Adults could presumably use this to infer that the evidence had been misleading and, later, that the informant was unreliable. This rich interpretation aligns with research showing that adults’ trust in an informant’s claims decreases when the informant’s statements are repeatedly revealed to be incorrect [39]. However, subjects may alternatively have read the X as a stronger version of the negation <the reward is *not* here>, as the X itself does not directly express higher-order content. In this lean interpretation, the X acts as an additional overrider emphasising the negation of the initial belief. Such quantitative and qualitative differences likely made it easier for participants in the Strong feedback condition to identify and assess the Unreliable informant’s evidence as misleading. The combined strength of multiple pieces of overriding evidence forms the basis for the generalisation, allowing individuals to acquire an undermining defeater like <the informant is unreliable>. Considering both the importance of the negated beliefs and the minimum strength they must have to ground the generalisation to <the informant if unreliable> helps to explain why some might fail to form the belief that the informant is unreliable, particularly in the Weak feedback condition. In some cases, subjects may fail to respond to the overriding evidence at all and thereby fail to form beliefs of the form <the reward is not there>. In other cases, subjects may form those negative beliefs but did not see them as strong enough collectively to justify the inference to <the source is unreliable>. Alternatively, participants may fail to grasp altogether the connection between the negative beliefs and the unreliability of the relevant source.

The results from the transfer trials also show differences related to the strength of the evidence. We expected that participants who had acquired an undermining defeater related to the Unreliable informant in the demonstration trials would show a preference for the Reliable over the Unreliable informant in the transfer trials. This was the case in the Strong feedback condition, in which participants preferred the Reliable informant in both transfer tasks. However, in the Weak feedback condition participants only showed a preference for the Reliable informant in the pointing trials. The difference observed in the screen choice trials (which were completed immediately after the demonstration trials) supports the idea that participants in the Strong feedback condition were responding to an undermining defeater related to the Unreliable informant and were able to apply this in a different context. Conversely, the pattern observed in the Weak feedback condition—where participants preferred the Reliable informant only in the pointing task—suggests that they may have learned about the Unreliable informant’s reliability during the screen choice trials rather than during the demonstration trials and then responded to this understanding in the pointing trials. Considering these findings with adults, along with research showing that children are sensitive to the strength of both initial evidence and counterevidence [26,28,29], it is evident that there are various ways in which overriding evidence may fail to achieve the collective strength required (relative to the initial positive evidence) to infer the unreliability of a source. Some of these possibilities were discussed above, but there may be others. The search for, and recognition of, these differences ought to inform future research investigating belief revision.

In an exploratory analysis, we looked at participants’ evidence following behaviour across the demonstration trials and categorised them based on their response patterns. Two distinct patterns emerged: 1) Discriminators, who differentiated between the Reliable and Unreliable informants, and 2) Followers, who followed the evidence of both informants. We were interested in whether these response patterns aligned with participants’ preferences for following the Reliable informant’s evidence in the transfer trials. We found that Discriminators preferred the Reliable informant in the transfer trials across both conditions, whereas Followers preferred the Reliable informant only in the Strong feedback condition, not in the Weak feedback condition. The remaining participants (“Others”) displayed no preference for the Reliable informant in either condition. These findings suggest a potentially important distinction between *acquiring* an undermining defeater and *responding* to it.

To assess whether someone has acquired an undermining defeater, in our study we must observe them respond to it. While the response to <the informant is unreliable>, strictly speaking, is something that happens at the level of judgment and thought, in experiments like the current one it can only be observed indirectly by the role that it plays in guiding action. Discriminators provide the clearest example of both acquiring and responding to an undermining defeater in relation to the Unreliable informant, initially following both informants, but later in the demonstration trials distinguishing between them and following the Unreliable informant less often. This pattern persisted in the transfer trials, where they maintained a preference for the Reliable informant. In contrast, Followers did not show clear evidence of having acquired an undermining defeater during demonstration trials, as they did not differentiate between the informants. However, in the transfer trials, their preference for the Reliable informant in the Strong feedback condition suggests that they could be responding to an undermining defeater, while no such preference was shown in the Weak feedback condition. Therefore, Followers in the Strong feedback condition may have acquired an undermining defeater during the demonstration trials, even though they did not respond to it at that stage. Rather, they may have refrained from responding to the undermining defeater in the demonstration trials because disregarding the Unreliable informant’s evidence offered no advantage in finding rewards. In fact, ignoring the evidence and choosing randomly may have required more effort than simply following, especially given the limited number of misleading trials. In the transfer trials, when informants were pit directly against one another, responding to the undermining defeater and favouring the Reliable informant became advantageous, as it led to more rewards, making it worthwhile to do so. Indeed, adults typically aim to maximise rewards in such situations [40]. It is possible that not all individuals in this group acquired an undermining defeater. Alternative hypotheses for following both informants in the demonstration trials include a lack of motivation to engage with the task or attention issues. These explanations also apply to the Followers in the Weak feedback condition who did not show a preference in the transfer trials. Thus, given the structure of the current task, we cannot conclude from the demonstration trials alone, if a participant did not discriminate between the Reliable and Unreliable informants, whether an undermining defeater was acquired. Instead, in such cases, we rely on the transfer trials, in which participants must choose between the informants, which provides them the opportunity to demonstrate whether they have made an inference about the informant’s reliability, are beneficial. Going forward, it will be important to consider whether paradigms such as this allow participants to demonstrate both the *acquisition of* and *response to* undermining defeaters.

In addition to the distinction between acquiring and responding to an undermining defeater, the differences between the conditions support our argument that the strength—either quantity, quality, or both—of the overriding evidence affects even adults’ ability to revise their beliefs. First, participants followed the Reliable informant’s evidence significantly more often than the Unreliable informant’s evidence in both the demonstration and transfer trials in the Strong feedback condition, but not the Weak feedback condition. Second, there were more Discriminators in the Strong feedback condition than the Weak feedback condition, and Followers only demonstrated a response to undermining defeaters in the transfer trials in the Strong feedback condition. Together these findings suggest that acquiring an undermining defeater through a generalisation over overriding defeaters is not trivial, and that the strength of the overriding evidence plays a crucial role; stronger evidence facilitates the acquisition of undermining defeaters. Though it does appear that overriding evidence needs to be stronger than the original evidence for a belief to be revised, it may still be possible to acquire an undermining defeater when the overriding evidence is not as strong. However, it is likely that this would require far more exposure to overriding evidence, and therefore in this type of paradigm far more trials. If so, this could help explain why most participants in the Weak feedback condition did not appear to acquire an undermining defeater, despite having the capacity to do so in principle, and why some participants were able to achieve it.

Overall, this study demonstrates that acquiring an undermining defeater through a generalisation over a series of overriding defeaters is not trivial, even for adults. We highlighted the important role played by the strength of overriding evidence—both in terms of quantity and quality—in acquiring an undermining defeater. When overriding evidence is stronger than the initial belief, adults can acquire an undermining defeater related to the unreliability of a source with minimal exposure to such evidence and can apply this to novel contexts. However, when overriding evidence is weaker, the likelihood of acquiring an undermining defeater reduces. Intuitively, this makes sense, as we are more likely to notice overriding evidence that has an impact on our daily lives; the clearer the reason to reject a previously held belief, the more likely we are to change our belief. If, as the results suggest, acquiring undermining defeaters depends on the strength of the overriding evidence, this should be accounted for when assessing capacities for acquiring and responding to similar undermining defeaters in non-linguistic populations. Additionally, our findings point to an important distinction between acquiring and responding to an undermining defeater. Future research should ensure that participants have opportunities to respond to the undermining defeater in contexts in which reliable and unreliable sources of evidence are pitted against one another and where such a response is the most appropriate.

## Supporting information

S1 Table Detailed outline of trial structure. (DOCX)

S1 File Supplementary results. (DOCX)
