# Supplementary material for: Adults show selective responses to unreliability based on the strength of counterevidence
Source: PLoS One. 2025 Nov 13;20(11):e0331480. doi: 10.1371/journal.pone.0331480 (PMC12614560; doi:10.1371/journal.pone.0331480)
Supplement: S1 Table — (DOCX) [file pone.0331480.s001.docx]

**Table S1. Detailed outline of trial structure**

|  | Demonstrator | Reliable Informant | Unreliable Informant |
| --- | --- | --- | --- |
| Demonstration Trials  (videos) | 4 x Crouching |  |  |
|  |  | 4 x Crouching |  |
|  |  |  | 6 x Crouching |
|  |  | 2 x Crouching |  |
|  | 2 x Crouching |  |  |
|  | 2 x Lifting |  |  |
|  |  | 4 x Lifting |  |
|  |  |  | 6 x Lifting |
|  |  | 2 x Lifting |  |
|  | 2 x Lifting |  |  |
|  | 2 x Sound |  |  |
|  |  |  | 4 x Sound |
|  |  | 4 x Sound |  |
| Transfer Trials (images) |  | 4 x Screen Choice | |
|  |  | 2 x Pointing | |
