# Supplementary material for: Adults show selective responses to unreliability based on the strength of counterevidence
Source: PLoS One. 2025 Nov 13;20(11):e0331480. doi: 10.1371/journal.pone.0331480 (PMC12614560; doi:10.1371/journal.pone.0331480)
Supplement: S1 File — (DOCX) [file pone.0331480.s002.docx]

# Supplementary results

##
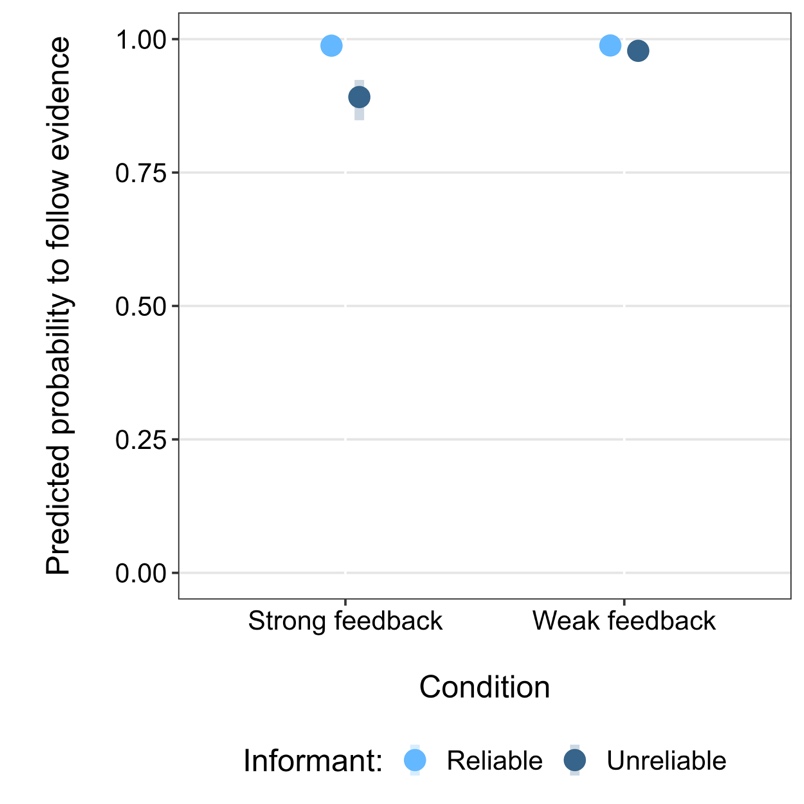
Demonstration trials

**Fig S1. Predicted probability of following each informant.** Predicted probability of participants in each condition following the evidence provided by each informant. Results are averaged over the effects of action side. Confidence intervals (95%) are shown for each informant-condition combination. As they are small for all but one point, they are more easily seen in Fig 3 of the main text, which provides a zoomed-in view.


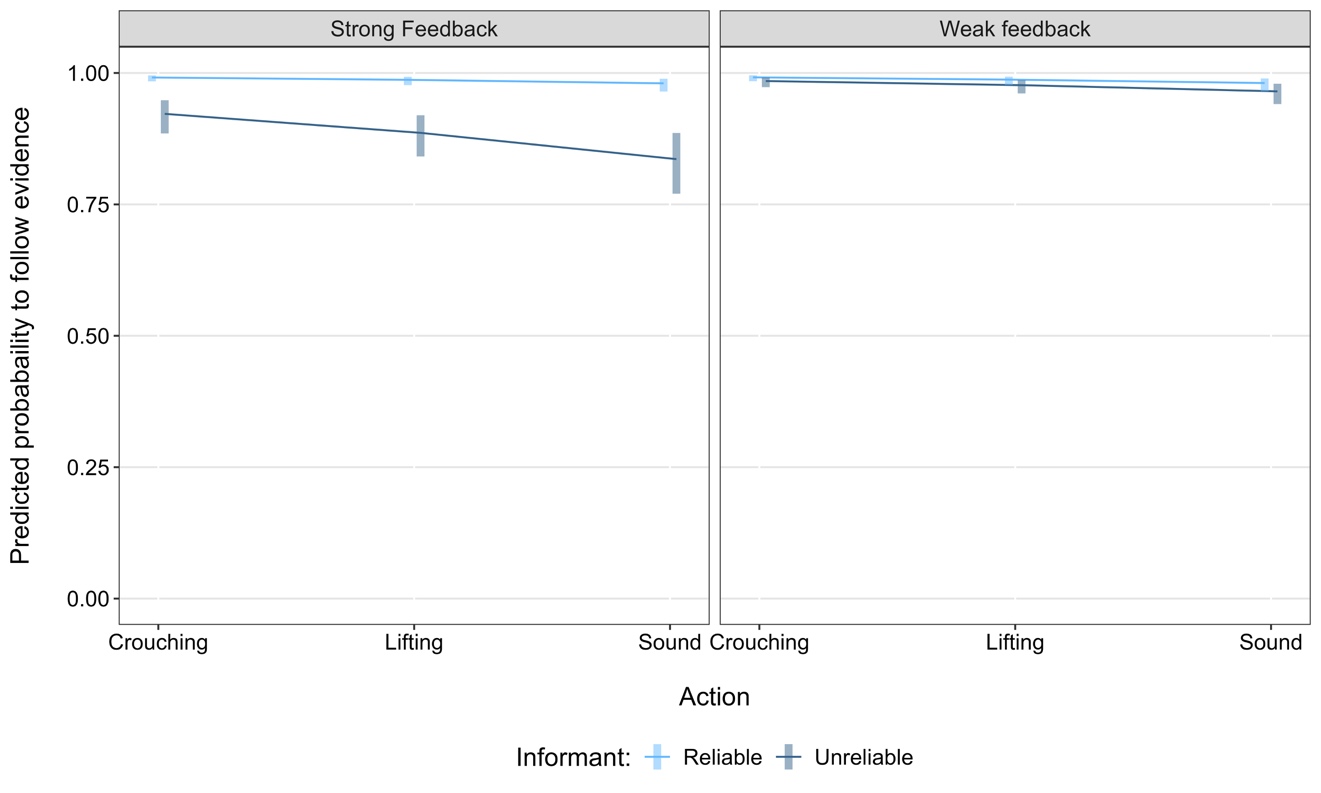
**Fig S2. Predicted probability of following each informant by condition.** Predicted probability of participants in the Strong feedback and Weak feedback conditions following the evidence provided by each informant across different actions. Confidence intervals (95%) are shown. Results are averaged over levels of action side.

## Categorising response patterns

As outlined in the main text, to be categorised as a “Discriminator” participants had to have followed the evidence in ≥75% of trials with the Reliable informant in all actions *and* of the Unreliable informant in ≥66% of the crouching trials, ≤84% of the Lifting trials, and ≤75% of the Sound trials (Fig S3). This means that they had to fit all six criteria to be categorised as such. The reason for the ≥75% criteria with the Reliable informant is that it encompasses following the evidence in at least five of the six crouching and lifting trials, and at least three of the four sound trials. The criteria for following the evidence of the Unreliable informant were necessarily different for each action. In the crouching trials, participants had to follow the evidence in at least four of the six trials. This level was set to capture participants who may have already discriminated between the informants in the crouching trials. In the lifting trials, participants had to follow the evidence in five or fewer of the six trials, this level was set to capture participants who had initially followed the evidence in all crouching trials meaning that following in five out of six trials would still be a reduction in following. Similarly, in the sound trials participants had to follow the evidence in three or fewer of the four trials. The remaining
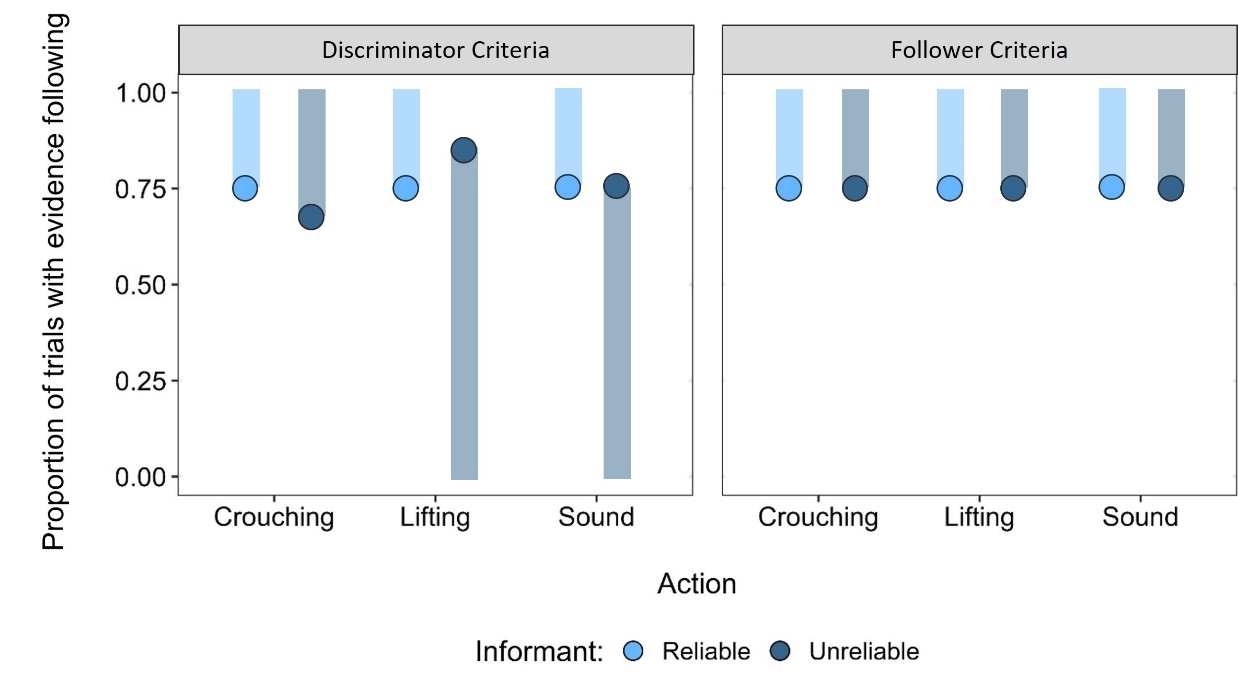
participants – “Others” – did not fit into either of these categories (Fig S4).

**Fig S3.** **Categorisation of participants as discriminators or followers.** Illustration of the range within which participants had to respond with each informant in each action to be categorised as a Discriminator or Follower.

**
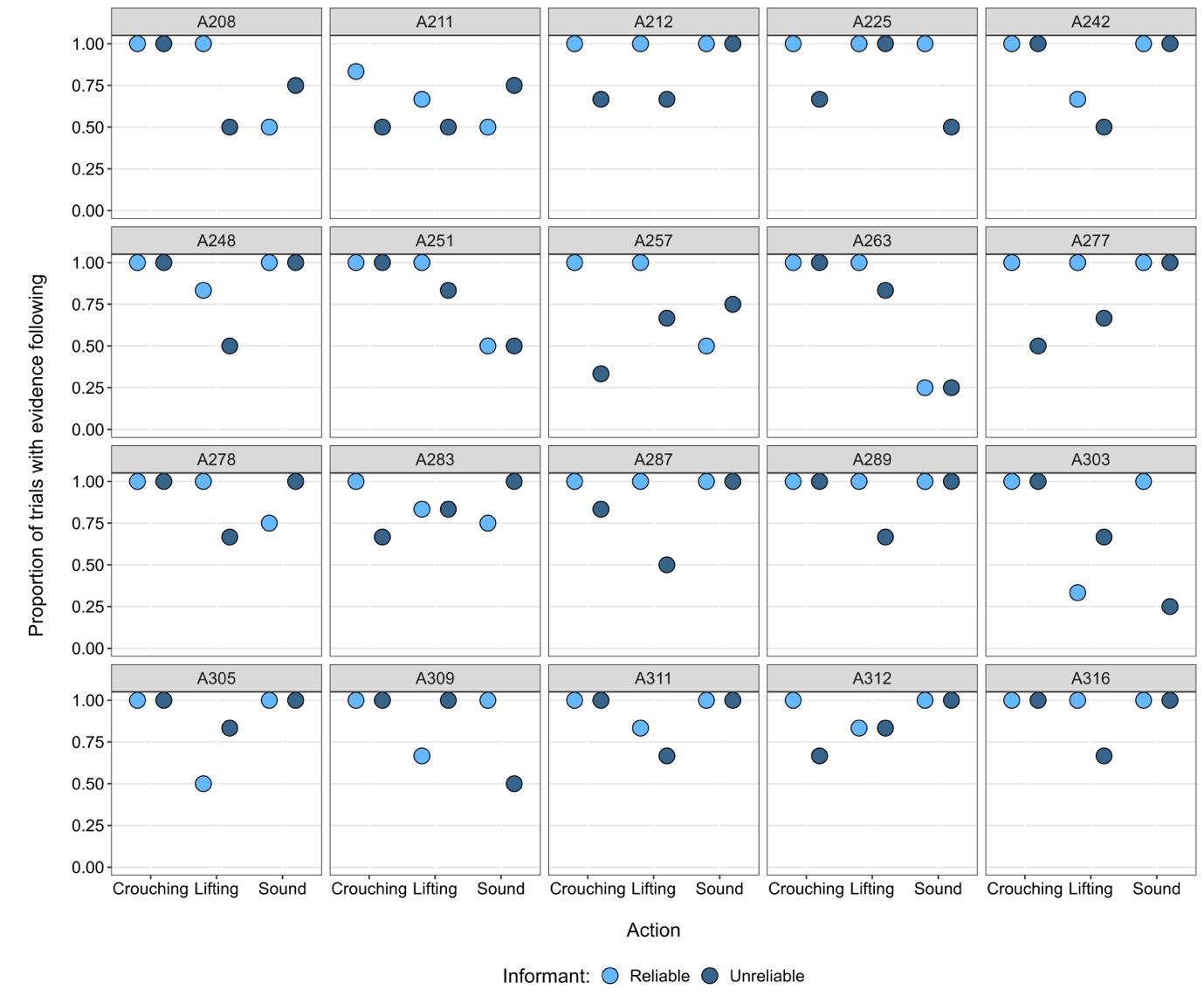
Fig S4. Individual demonstration trials response patterns of participants categorised as Others.**

Table S2. T-tests against chance (50%) for following the evidence of the Reliable informant in the four Screen Choice transfer trials for each response category in each condition.

|  | **Condition** | | | | | |
| --- | --- | --- | --- | --- | --- | --- |
|  | **Strong feedback** | | | **Weak feedback** | | |
| **Category** | ***n*** | ***M*** | ***p*** | ***n*** | ***M*** | ***p*** |
| Discriminators | 20 | 2.60 | .012 | 5 | 1.8 | .813 |
| Followers | 28 | 2.32 | .071 | 47 | 1.79 | .885 |
| Others | 12 | 2.08 | .410 | 8 | 1.88 | .618 |

Table S3. T-tests against chance (50%) for following the evidence of the Reliable informant in the two Pointing transfer trials for each response category in each condition.

|  | **Condition** | | | | | |
| --- | --- | --- | --- | --- | --- | --- |
|  | **Strong feedback** | | | **Weak feedback** | | |
| **Category** | ***n*** | ***M*** | ***p*** | ***n*** | ***M*** | ***p*** |
| Discriminators | 20 | 1.25 | .117 | 5 | 1.8 | .008 |
| Followers | 28 | 1.46 | .002 | 47 | 1.11 | .179 |
| Others | 12 | 1.08 | .377 | 8 | 1.13 | .342 |
